# Ferroptosis affects cognitive dysfunction and the progression of mental illnesses

**DOI:** 10.1192/j.eurpsy.2024.1556

**Published:** 2024-08-27

**Authors:** B. Nycz, K. Krysta

**Affiliations:** Department of Rehabilitation Psychiatry, Medical University of Silesia, Katowice, Poland

## Abstract

**Introduction:**

Ferroptosis is a programmed form of cell death characterized by excessive accumulation of intracellular iron fraction, uncontrolled lipid peroxidation, impairment of glutathione-dependent antioxidant functions, and loss of oxidative-antioxidant balance. Nerve cells are sensitive to excessive amounts of iron, which impairs the functioning of mitochondria and leads to their death. Ferroptosis has been identified in neurological diseases such as stroke, Alzheimer’s disease, Parkinson’s disease. Features of ferroptotic cells have been observed in models expressing cognitive deficits, and ferroptosis-related genes have been associated with the development of mental illnesses.

**Objectives:**

The aim of the study was to analyze the available literature on the relationship between the occurrence of ferroptosis and cognitive impairment occurring in mental diseases, such as schizophrenia

**Methods:**

The publications found in the PubMed database were analyzed after entering the following entries: ferroptosis, mental illness, cognitive functions, schizophrenia.

**Results:**

Ferroptosis occurs in mental illnesses. Increased expression of the TP53 and VEGFA genes, which are associated with ferroptosis, has been identified in patients suffering from schizophrenia. Animal research confirms that disturbed iron homeostasis causes iron overload in nerve cells, which leads to ferroptosis and has a neurodegenerative effect, as well as deepens cognitive deficits. The use of iron chelator has a neuroprotective effect and reduces the occurrence of cognitive disorders.

**Conclusions:**

Genes associated with ferroptosis may influence the development of schizophrenia, which means that ferroptosis may be involved in the pathophysiology of schizophrenia. Excess iron inside nerve cells, as a feature of ferroptosis, may affect the deterioration of cognitive functioning. Administration of iron chelators protects neurons by reducing the toxic effects of iron.

**Disclosure of Interest:**

None Declared

